# Cardiac damage in patients with the severe type of coronavirus disease 2019 (COVID-19)

**DOI:** 10.1186/s12872-020-01758-w

**Published:** 2020-11-10

**Authors:** Jing Li, Yinghua Zhang, Fang Wang, Bing Liu, Hui Li, Guodong Tang, Zhigang Chang, Aihua Liu, Chunyi Fu, You Lv, Jing Gao, Jing Li

**Affiliations:** 1 Division of Cardiology, Beijing Hospital, #1 Dahua Road, Dongcheng District, Beijing, 100730 China; 2 Division of Cardiology, Xuanwu Hospital Capital Medical University, #45 Changchun Street, Xicheng District, Beijing, 100053 China; 3 Division of Intensive Care Unit, Beijing Hospital, Beijing, 100730 China; 4 Division of Rheumatology and Immunology, Beijing Hospital, Beijing, 100730 China; 5 Division of Emergency, Beijing Hospital, Beijing, 100730 China; 6 National Center Research Center of Geriatric Diseases [Xuanwu Hospital], Beijing, 100053 China

**Keywords:** Coronavirus, Severe pneumonia, Cardiac damage

## Abstract

**Background:**

Coronavirus disease 2019 (COVID-19) has become a global pandemic. Studies showed COVID-19 affected not only the lung but also other organs. In this study, we aimed to explore the cardiac damage in patients with COVID-19.

**Methods:**

We collected data of 100 patients diagnosed as severe type of COVID-19 from February 8 to April 10, 2020, including demographics, illness history, physical examination, laboratory test, and treatment. In-hospital mortality were observed. Cardiac damage was defined as plasma hypersensitive troponin I (hsTnI) over 34.2 pg/ml and/or N-terminal-pro brain natriuretic peptide (NTproBNP) above 450 pg/ml at the age < 50, above 900 pg/ml at the age < 75, or above 1800 pg/ml at the age ≥ 75.

**Results:**

The median age of the patients was 62.0 years old. 69 (69.0%) had comorbidities, mainly presenting hypertension, diabetes, and cardiovascular disease. Fever (69 [69.0%]), cough (63 [63.0%]), chest distress (13 [13.0%]), and fatigue (12 [12.0%]) were the common initial symptoms. Cardiac damage occurred in 25 patients. In the subgroups, hsTnI was significantly higher in elder patients (≥ 60 years) than in the young (median [IQR], 5.2 [2.2–12.8] vs. 1.9 [1.9–6.2], *p* = 0.018) and was higher in men than in women (4.2 [1.9–12.8] vs. 2.9 [1.9–7.4], *p* = 0.018). The prevalence of increased NTproBNP was significantly higher in men than in women (32.1% vs. 9.1%, *p* = 0.006), but was similar between the elder and young patients (20.0% vs. 25.0%, *p* = 0.554). After multivariable analysis, male and hypertension were the risk factors of cardiac damage. The mortality was 4.0%.

**Conclusions:**

Cardiac damage exists in patients with the severe type of COVID-19, especially in male patients with hypertension. Clinicians should pay more attention to cardiac damage.

## Introduction

Since the novel coronavirus–severe acute respiratory syndrome coronavirus 2 (SARS-CoV-2) named by the Coronavirus Study Group of the International Committee on Taxonomy of Viruses [1], was discovered in December 2019, it quickly spread throughout China and other countries [2–5]. As of April 30, 2020, SARS-CoV-2 has broken out in 213 countries, areas and territories with 3,090,445 confirmed cases and 217,769 deaths [6]. Coronavirus disease 2019 (COVID-19) caused by SARS-CoV-2 could be classified into four clinical types: mild, moderate, severe and critical types [7]. More than 80% are mild or moderate with relatively good short-term prognosis due to the self-limiting process according to the study with 44,672 confirmed COVID-19 cases released by the Chinese Center for Disease Control and Prevention on February 17, 2020 [8]. However, 6,168 (13.8%) cases belonged to the severe type and were more likely to develop into the critical type followed by a death rate of 49%, far beyond the average mortality of 2.3%.

Researchers have reported that patients with COVID-19 had acute cardiac injury, which is associated with a higher risk of in-hospital mortality [4, 9, 10]. However, studies focusing on cardiac damage in patients with the severe COVID-19 are few.

In this study, we aim to investigate the clinical findings and cardiac damage in patients with the severe type of COVID-19, and hope to contribute to the prevention and treatment.

## Methods

### Study population

We retrospectively collected data of all 100 patients at the Sino-French New Town area of Tongji Hospital, Wuhan, where was aided and charged by the medical team of Beijing Hospital from February 8 to April 10, 2020. Patients were all diagnosed as COVID-19 and classified into the severe type according to the Diagnosis and Treatment of Pneumonia Infected by Novel Coronavirus (5th trial edition) pressed by the General Office of the National Health Commission and the General Office of the National Administration of Traditional Chinese Medicine [7]. Severe type met at least one of the following criteria: (1) dyspnea, respiratory frequency ≥ 30/minute, (2) blood oxygen saturation ≤ 93% at rest, (3) PaO2/FiO2 ratio ≤ 300. Critical type met at least one of the following criteria: (1) respiratory failure with mechanical ventilation, (2) septic shock, (3) transferred to the intensive care unit due to multiple organ failure. Patients with acute coronary syndrome or acute heart failure at admission or in the latest one month were excluded. This study was approved by the Ethics Commission of Beijing Hospital (2020BJYYEC-035–01).

Laboratory confirmation of SARS­CoV-2 was done by real-time RT-PCR. The protocol was the same as the document published recently [4]. We also examined other respiratory viruses with real-time RT-­PCR, including influenza A virus (H1N1, H3N2, H7N9), influenza B virus, respiratory syncytial virus, parainfluenza virus, adenovirus, SARS coronavirus (SARS-­CoV), and MERS coronavirus (MERS­-CoV). Sputum or endotracheal aspirates were obtained at admission for the identification of possible causative bacteria or fungi.

### Data collection

We obtained demographic, illness history, physical examination, laboratory test, management, and outcome data from patients’ medical records. Blood oxygen saturation was measured after oxygen therapy. In-hospital mortality were observed.

Laboratory tests were conducted within 24 h after admission, including a complete blood count, procalcitonin, interleukin-6, ferritin, coagulation profile, renal and liver function, hypersensitive troponin I (hsTnI), and N-terminal-pro brain natriuretic peptide (NTproBNP).

### Definition of cardiac damage

Cardiac damage was defined as plasma hsTnI over 34.2 pg/ml and/or NTproBNP above 450 pg/ml at the age age < 50, above 900 pg/ml at the age < 75, or above 1800 pg/ml at the age ≥ 75 [11].

### Statistical analysis

Continuous variables were expressed as mean ± SD when they were normally distributed or median (IQR) when they were not, and compared with the *t-*test or Mann–Whitney U test, respectively; categorical variables were expressed as number (%) and compared by χ^2^ test or Fisher’s exact test. Logistic regression analysis was performed to identify variables with a significant independent association with cardiac damage. Demographics (age and sex), potential confounders (hypertension, diabetes, cardiovascular disease, and hyperlipidemia), and variables with p ≤ 0.05 in the univariate analysis were adjusted. A two-sided α of less than 0.05 was considered statistically significant. Statistical analyses were done using the SPSS software (version 23) for all analyses.

## Results

From February 8 to April 10, 2020, 100 laboratory-confirmed COVID-19 patients were classified as the severe type at the Sino-French New Town area, with an median age of 62.0 years old. 56 (56.0%) cases were men. 69.0% of the patients had comorbidities, of which hypertension, diabetes, and cardiovascular disease were the top three diseases. The initial symptoms were mainly fever, cough, chest distress, and fatigue. Ten patients had oxygen saturation below 93% after nasal oxygen supply at admission (Table 1).

**Table 1:** Demographics and clinical characteristics of severe COVID-19

Variables	Median (IQR), or N (%)
	Patients (n = 100)
Age, y	
Median (IQR)	62.0 (51.0–70.8)
< 60	40 (40.0)
≥ 60	60 (60.0)
Sex	
Male	56 (56.0)
Female	44 (44.0)
Comorbidities	69 (69.0)
Hypertension	40 (40.0)
Diabetes	21 (21.0)
CVD	15 (15.0)
COPD	12 (12.0)
Malignancy	13 (13.0)
Hypothyroidism	2 (2.0)
Liver cirrhosis	3 (3.0)
Hyperlipidemia	2 (2.0)
Anemia	2 (2.0)
Initial symptoms	
Fever	69 (69.0)
Cough	63 (63.0)
Chest distress	13 (13.0)
Fatigue	12 (12.0)
Sputum	7 (7.0)
Myalgia	5 (5.0)
Dyspnea	6 (6.0)
Headache	4 (4.0)
Sore throat	4 (4.0)
Chest pain	2 (2.0)
Diarrhea	3 (3.0)
Nausea	3 (3.0)
SBP, mmHg	135.0 (122.0–149.0)
DBP, mmHg	81.5 (74.8–91.0)
Heart rate, bpm	92.5 (79.5–103.3)
SPO2, %^§^	97.0 (95.0–98.0)
≤ 93.0	10 (10.0)
Days from illness onset to admission	14.0 (7.0–28.0)
Hospitalization time, day	21.0 (15.0–39.5)

^§^Oxygen saturation was measured on admission after receiving oxygen therapy. COVID-19 = coronavirus disease 2019; COPD = chronic obstructive pulmonary disease; CVD = cardiovascular disease; SBP = systolic blood pressure; DBP = diastolic blood pressure

Cardiac damage occurred in 25 patients. (Table 2) Increased hsTnI was in 9 (9.0%) patients; increased NTproBNP was in 22 (22.0%) patients. Subgroup analysis showed that significant age and sex differences in hsTnI. (Tables 3, 4) The elderly patients had higher plasma hsTnI levels, so did the males, who also had significantly higher NTproBNP levels than the females. Although NTproBNP level was far higher in the elder, the statistical difference disappeared when taking the effect of age on NTproBNP into consideration.

**Table 2:** Laboratory characteristics of severe COVID-19

Variables	Mean ± SD, Median (IQR), or N (%)
White blood count, × 10^9^/L	5.8 (4.3–8.5)
Neutrophil count, × 10^9^/L	3.7 (2.4–7.1)
Lymphocyte count, × 10^9^/L	1.1 (0.7–1.6)
Platelet count, × 10^9^/L	211.5 (164.0–301.0)
Hemoglobin, g/L	119.2 ± 20.2
Prothrombin time, s	13.9 (13.4–14.4)
Activated partial thromboplastin time, s	39.1 (35.7–43.5)
Fibrinogen, g/L	4.76 ± 1.55
D-dimer, mg/L	1.1 (0.4–3.5)
Alanine aminotransferase, U/L	19.0 (12.0–41.0)
Creatinine, μmol/L	67.0 (57.5–86.5)
Procalcitonin, ng/ml	0.07 (0.03–0.10)
Interleukin-6, pg/ml	7.67 (3.01–23.51)
Ferritin, ng/ml	559.3 (304.7–1214.6)
hsCRP, mg/ml	11.6 (2.6–47.1)
Cardiac damage	25 (25.0)
hsTnI, pg/ml	3.5 (1.9–11.2)
Increased (> 34.2)	9 (9.0)
NTproBNP, pg/ml	133.5 (42.3–369.5)
Increased^#^	22 (22.0)
Influenza A antibody	5 (5.0)

COVID-19 = coronavirus disease 2019; hsCRP = hypersensitive C-reactive protein; hsTnI = hypersensitive troponin I; NTproBNP = N-terminal-pro brain natriuretic peptide. ^#^Increased NTproBNP was above 450 pg/ml at the age < 50, above 900 pg/ml at the age < 75, or above 1800 pg/ml at the age ≥ 75

**Table 3:** Age differences in cardiac damage in patients with severe type of COVID-19

	Median (IQR) or N (%)		*p* value^a^
	Age ≥ 60 y (n = 60)	Age < 60 y (n = 40)	
Female	29 (48.3)	15 (37.5)	0.285
Comorbidities	48 (80.0)	21 (52.5)	0.004
Hypertension	29 (48.3)	11 (27.5)	0.037
Diabetes	17 (28.3)	4 (10.0)	0.027
CVD	9 (15.0)	6 (15.0)	1.000
Hyperlipidemia	2 (3.3)	0	0.515
hsTnI, pg/ml	5.2 (2.2–12.8)	1.9 (1.9–6.2)	0.018
Increased (> 34.2)	7 (12.7)	3 (8.3)	0.755
NTproBNP, pg/ml	177.5 (97.5–369.5)	55.5 (18.0–432.8)	0.018
Increased^#^	12 (20.0)	10 (25.0)	0.554

^a^*p* < .05 was considered statistically significant. COVID-19 = coronavirus disease 2019; CVD = cardiovascular disease; hsTnI = hypersensitive troponin I; NTproBNP = N-terminal-pro brain natriuretic peptide. ^#^Increased NTproBNP was above 450 pg/ml at the age < 50, above 900 pg/ml at the age < 75, or above 1800 pg/ml at the age ≥ 75

**Table 4:** Sex differences in cardiac damage in patients with severe type of COVID-19

	Median (IQR) or N (%)		*p* Value ^a^
	Men (n = 56)	Women (n = 44)	
Age ≥ 60 y	31 (55.4)	29 (65.9)	0.285
Comorbidities	34 (60.7)	35 (79.5)	0.043
Hypertension	18 (32.1)	22 (50.0)	0.070
Diabetes	9 (16.1)	12 (27.3)	0.172
CVD	5 (8.9)	10 (22.7)	0.055
Hyperlipidemia	1 (1.8)	1 (2.3)	1.000
Cardiac damage	18 (32.1)	7 (15.9)	0.063
hsTnI, pg/ml	4.2 (1.9–12.8)	2.9 (1.9–7.4)	0.018
Increased (> 34.2)	5 (10.4)	5 (11.6)	0.340
NTproBNP, pg/ml	272.5 (57.0–559.8)	86.0 (31.3–209.3)	0.013
Increased^#^	18 (32.1)	4 (9.1)	0.006

^a^*p* < .05 was considered statistically significant. COVID-19 = coronavirus disease 2019; CVD = cardiovascular disease; hsTnI = hypersensitive troponin I; NTproBNP = N-terminal-pro brain natriuretic peptide. ^#^Increased NTproBNP was above 450 pg/ml at the age < 50, above 900 pg/ml at the age < 75, or above 1800 pg/ml at the age ≥ 75

Patients with cardiac damage had a higher proportion of hypertension and diabetes, compared to those without cardiac damage. White blood count, prothrombin time, d-dimer, creatinine, interleukin-6, procalcitonin, and hsCRP levels were significantly different between the two groups. After adjusting for age, sex, hypertension, diabetes, cardiovascular disease, hyperlipidemia, and variables with significant differences, we found male and hypertension were the risk factors of cardiac damage in patients with severe COVID-19. (Tables 5, 6).

**Table 5:** Demographics and clinical characteristics of patients with and without cardiac damage

Variables	Mean ± SD, median (IQR), or N (%)		*p* Value
	Cardiac damage (n = 25)	Non cardiac damage (n = 75)	
Age, y	71.0 (52.0–79.5)	62.0 (49.0–67.0)	0.084
Gender			0.063
Male	18 (72.0)	38 (50.7)	
Female	7 (28.0)	37 (49.3)	
Comorbidities	21 (84.0)	48 (64.0)	0.061
Hypertension	18 (72.0)	22 (29.3)	0.000
Diabetes	9 (36.0)	12 (16.0)	0.033
CVD	7 (28.0)	8 (10.7)	0.075
COPD	2 (8.0)	10 (13.3)	0.722
Malignancy	5 (20.0)	8 (10.7)	0.391
Hypothyroidism	1 (4.0)	1 (1.3)	0.439
Liver cirrhosis	0	3 (4.0)	0.571
Hyperlipidemia	1 (4.0)	1 (1.3)	0.439
Anemia	2 (8.0)	0	0.061
SBP, mmHg	135.0 (125.0–151.0)	134.0 (121.0–148.5)	0.368
DBP, mmHg	82.0 (76.0–90.0)	81.0 (72.5–92.0)	0.596
Heart Rate, bpm	89.3 ± 16.9	92.9 ± 16.3	0.351
SPO2 ≤ 93.0%^§^	5 (20.0)	5 (6.7)	0.124
Days from illness onset to admission	10.0 (4.0–23.5)	14.0 (8.0–28.0)	0.152
Hospitalization time, day	30.0 (18.0–50.0)	19.0 (12.8–36.5)	0.024
White blood count, × 10^9^/L	7.6 (4.6–10.5)	5.5 (4.0–7.3)	0.020
Lymphocyte count, × 10^9^/L	0.9 (1.3–0.7)	1.2 (0.8–1.7)	0.286
Platelet count, × 10^9^/L	190.0 (156.0–304.0)	227.0 (170.0–297.0)	0.389
Hemoglobin, g/L	115.1 ± 27.3	120.5 ± 17.4	0.381
Prothrombin time, s	14.3 (13.5–15.4)	13.9 (13.3–14.2)	0.029
Activated partial thromboplastin time, s	39.2 (37.2–45.4)	39.1 (35.7–42.2)	0.566
Fibrinogen, g/L	4.88 ± 1.70	119.2 ± 20.2	0.681
D-dimer, mg/L	2.44 (1.03–8.29)	1.01 (0.40–2.69)	0.008
Alanine aminotransferase, U/L	25.5 (12.0–44.0)	19.0 (12.0–38.8)	0.463
Creatinine, μmol/L	79.0 (64.0–94.8)	64.0 (55.0–80.5)	0.022
Procalcitonin, ng/ml	0.08 (0.05–0.23)	0.06 (0.03–0.09)	0.013
Interleukin-6, pg/ml	26.23 (6.09–46.06)	6.08 (2.54–14.15)	0.002
Ferritin, ng/ml	669.2 (332.9–992.9)	457.6 (303.3–1292.6)	0.773
hsCRP, mg/ml	39.4 (10.2–79.6)	8 (2.4–41.7)	0.019
Influenza A antibody	1 (4.0)	4 (5.3)	1.000
Death	0	4 (5.3)	0.556

^§^Oxygen saturation was measured on admission after receiving oxygen therapy. COVID-19 = coronavirus disease 2019; COPD = chronic obstructive pulmonary disease; CVD = cardiovascular disease; SBP = systolic blood pressure; DBP = diastolic blood pressure; hsCRP = hypersensitive C-reactive protein

**Table 6:** Logistic multivariable models for determinants of cardiac damage

Variables	β	Adjusted HR	95% CI	*p* Value
Age	− 0.01	0.99	0.93–1.06	0.771
Male	1.63	5.09	1.19–22.17	0.028
Hypertension	2.29	9.88	2.52–28.70	0.001
Diabetes	0.90	2.46	0.36–17.00	0.360
Cardiovascular disease	1.32	3.73	0.41–33.84	0.242
Hyperlipidemia	1.46	4.32	0.04–530.45	0.551
White blood count	− 0.04	0.96	0.79–1.18	0.725
Prothrombin time	0.17	1.18	0.75–1.86	0.468
d-dimer	0.16	1.18	0.97–1.43	0.090
Creatinine	0.01	1.01	0.99–1.04	0.309
Interleukin-6	− 0.01	0.99	0.98–1.00	0.994
Procalcitonin	0.91	2.49	0.14–43.54	0.531
hsCRP	0.02	1.02	1.00–1.04	0.125

Adjusted for age, sex, hypertension, diabetes, cardiovascular disease, hyperlipidemia, white blood count, prothrombin time, d-dimer, creatinine, interleukin-6, procalcitonin, and hsCRP

By the end of April 10, 2020, four (4.0%) patients died. In the same period, 96 patients, of which 2 cases deteriorated to critical type but ultimately recovered and discharged from the hospital according to the Criteria of Diagnosis and Treatment of Pneumonia Infected by Novel Coronavirus (5th trial edition). (Table 7).

**Table 7:** Treatment and outcomes for severe COVID-19

Variables	N (%)
Treatment	
Antiviral therapy	92 (92.0)
Antibiotic therapy	35 (35.0)
Traditional Chinese medicine	62 (62.0)
Clinical outcomes	
Discharge	94 (94.0)
Death	4 (4.0)

COVID-19 = coronavirus disease 2019

## Discussion

In this retrospective study, we analyzed data from 100 patients with severe type of laboratory-confirmed COVID-19. Fever, cough, chest distress, and fatigue were common symptoms. Patients with severe COVID-19 also presented lymphopenia, elevated interleukin-6, procalcitonin, and D-dimer. These were consistent with recent researches [3, 4, 12, 13]. More than half of the patients had comorbidities, mainly including hypertension, diabetes, and cardiovascular disease. The prevalence of cardiac damage was 25%. The mortality of severe COVID-19 was 4%.

Huang et al. reported acute cardiac and kidney injuries in COVID-19 patients. In our study, one-quarter of the patients had cardiac damage, suggesting COVID-19 was a systemic disease and SARS-CoV-2 could cause multiorgan damage. The mechanism of cardiac damage resulted from SARS-CoV-2 is unclear. We consider two possible explanations: (1) immune response elicited by the coronavirus may lead to systemic inflammatory response [14, 15]; (2) the virus exists in multiple organ systems to attack tissues [16].

The prevalence of elevated NTproBNP was 22.0% in our study, which was rarely reported previously. Published studies showed that the incidence of myocardial injury presenting elevated hsTnI was 7.2–19.7% in general COVID-19 patients and 23% in critical ill patients, higher than 9.0% in our study [9, 10, 17]. It possibly owes to different criteria of elevated hsTnI, sample sizes, and the phases of COVID-19 breakout.

In subgroups, there was no significant age or sex difference in cardiac damage, but a higher proportion of comorbidities, especially hypertension and diabetes, existed in patients with cardiac damage. After multivariable analysis, we found males and hypertension were the risk factors of cardiac damage. It is consistent with Shi’s report [10]. As all the patients in our study didn’t have an acute coronary syndrome or acute heart failure at admission or in the latest one month, it may suggest that male patients with hypertension are more susceptible to cardiac damage. The possible mechanism is that hypertension-induced cardiac damage is associated with mitochondrial injury, which can be caused by SARS-COV-2 [18, 19]. Estrogen may also play an important protective role in the process [20]. Researchers found cardiac troponins elevation was associated with the male [21, 22].

As of April 10, 96% of the patients were discharged from the hospital. The mortality was 4%, less than the average mortality of severe acute respiratory syndrome (SARS) and Middle Eastern respiratory syndrome (MERS), which were 11% and 35% [23, 24]. It indicates the prognosis of COVID-19 is generally good. There was no difference in mortality between patients with cardiac damage and those without (0 vs. 5.3%, *p* = 0.556).

### Limitations

Our study has several limitations. First, it’s a study with a small sample size, confounding factors and selection bias are inevitable. Second, we had no data on medication history, electrocardiography, and echocardiography and we can’t describe and discuss the results adequately. Third, we didn’t include the outcomes after patients were discharged from the hospital.

## Conclusion

COVID-19 is a systemic disease. Cardiac damage exists in patients with the severe type of COVID-19, especially in male patients with hypertension. Clinicians should pay more attention to cardiac damage. Further studies with large sample size are needed to verify our findings.

## Data Availability

The datasets used and/or analysed during the current study are available from the corresponding author on reasonable request.

## Abbreviations

SARS-CoV-2: Severe acute respiratory syndrome coronavirus 2
COVID-19: Coronavirus disease 2019;
SARS­-CoV: SARS coronavirus
MERS-­CoV: MERS coronavirus
hsTnI: Hypersensitive troponin I
NTproBNP: N-terminal-pro brain natriuretic peptide
